# Male genitourinary schistosomiasis-related symptoms among long-term Western African migrants in Spain: a prospective population-based screening study

**DOI:** 10.1186/s40249-024-01190-8

**Published:** 2024-03-07

**Authors:** Sílvia Roure, Xavier Vallès, Olga Pérez-Quílez, Israel López-Muñoz, Anna Chamorro, Elena Abad, Lluís Valerio, Laura Soldevila, Sergio España, Alaa H. A. Hegazy, Gema Fernández-Rivas, Ester Gorriz, Dolores Herena, Mário Oliveira, Maria Carme Miralles, Carmen Conde, Juan José Montero-Alia, Elia Fernández-Pedregal, Jose Miranda-Sánchez, Josep M. Llibre, Mar Isnard, Josep Maria Bonet, Oriol Estrada, Núria Prat, Bonaventura Clotet, The Schisto-Stop study group, Montserrat Riera, Núria Rovira, Ainhoa López, Mayra Segura, Susana Escoda, Janeth Karin Villalaz-Gonzales, Maria Jesús Delgado, Iciar Ferre-García, Sandra Santamaria, Marilen Matero

**Affiliations:** 1 International Health Program (PROSICS), Barcelona North Metropolitan Territorial Directorate for Infectious Diseases, Catalan Institute for Health, CAP La Salut, Passatge dels Encants S/N, 08916 Badalona, Spain; 2 Fundació Lluita contra les Infeccions, c/ Can Ruti s/n, 08916 Badalona, Spain; 3 Infectious Diseases Department, Hospital Universitari Germans Trias i Pujol, c/ Can Ruti s/n, 08916 Badalona, Spain; 4 Germans Trias i Pujol Research Institute, c/ Can Ruti s/n, 08916 Badalona, Spain; 5 Microbiology Department, Germans Trias i Pujol University Hospital, c/ Can Ruti s/n, 08916 Badalona, Spain; 6 Department of Genetics and Microbiology, Universitat Autònoma de Barcelona, c/ Can Ruti s/n, 08916 Badalona, Spain; 7 Faculty of Medicine, University of Asyut, El Fateh, Assiut Governorate, 71515 Egypt; 8 Department of Urology, Germans Trias i Pujol University Hospital, c/ Can Ruti s/n, 08916 Badalona, Spain; 9 Canovelles Primary Health Care Unit, Barcelona North Metropolitan Health Directorate, Catalan Institute for Health, C/ Indústria 23, 08420 Canovelles, Spain; 10 Primary Health Care Unit Mataró-3 (Rocafonda-Palau), Barcelona North Metropolitan Health Directorate, Catalan Institute for Health, Camí Ral el Ravalet 208, Mataró, 08302 Barcelona, Spain; 11 Barcelona North Metropolitan Primary Care Directorate, Catalan Institute for Health, Ctra. de Barcelona 473, Sabadell, 08204 Barcelona, Spain; 12 Directorate for Innovation and Interdisciplinary Cooperation, Barcelona North Metropolitan Health Directorate, Catalan Institute for Health, C/ Can Ruti S/N, 08916 Badalona, Spain; 13 IrsiCaixa-AIDS Research Institute, Hospital Universitari Germans Trias i Pujol University Hospital, c/ Can Ruti s/n, 08916 Badalona, Spain; 14 Infectious Diseases Directorate, Barcelona North Metropolitan Health Directorate, Catalan Institute for Health, C/ Can Ruti S/N, 08916 Badalona, Spain

**Keywords:** Schistosomiasis, Chronic schistosomiasis, Urogenital schistosomiasis, Male genital schistosomiasis, Long-term migrant

## Abstract

**Background:**

Schistosomiasis is highly endemic in sub-Saharan Africa and frequently imported to Europe. Male urogenital manifestations are often neglected. We aimed to ascertain the prevalence of genitourinary clinical signs and symptoms among long-term African migrants in a non-endemic European country using a serology test.

**Methods:**

We carried out a prospective, community-based cross-sectional study of adult male migrants from sub-Saharan Africa living in Spain. *Schistosoma* serology tests and microscopic urine examinations were carried out, and clinical data were obtained from an electronic medical record search and a structured questionnaire.

**Results:**

We included 388 adult males, mean age 43.5 years [Standard Deviation (*SD)* = 12.0, range: 18–76]. The median time since migration to the European Union was 17 [Interquartile range (IQR): 11–21] years. The most frequent country of origin was Senegal (*N* = 179, 46.1%). Of the 338, 147 (37.6%) tested positive for *Schistosoma.* Parasite eggs were present in the urine of only 1.3%. Nine genitourinary clinical items were significantly associated with positive *Schistosoma* serology results: pelvic pain (45.2%; *OR* = 1.57, 95% *CI*: 1.0–2.4), pain on ejaculation (14.5%; *OR* = 1.85, 95% *CI*: 1.0–3.5), dyspareunia (12.4%; *OR* = 2.45, 95% *CI*: 1.2–5.2), erectile dysfunction (9.5%; *OR* = 3.10, 95% *CI*: 1.3–7.6), self-reported episodes of infertility (32.1%; *OR* = 1.69, 95% *CI*: 1.0–2.8), haematuria (55.2%; *OR* = 2.37, 95% *CI*: 1.5–3.6), dysuria (52.1%; *OR* = 2.01, 95% *CI*: 1.3–3.1), undiagnosed syndromic STIs (5.4%), and orchitis (20.7%; *OR* = 1.81, 95% *CI*: 1.0–3.1). Clinical signs tended to cluster.

**Conclusions:**

Urogenital clinical signs and symptoms are prevalent among male African long-term migrants with a positive *Schistosoma* serology results. Genital involvement can be frequent even among those with long periods of non-residence in their sub-Saharan African countries of origin. Further research is needed to develop diagnostic tools and validate therapeutic approaches to chronic schistosomiasis.

**Graphical Abstract:**

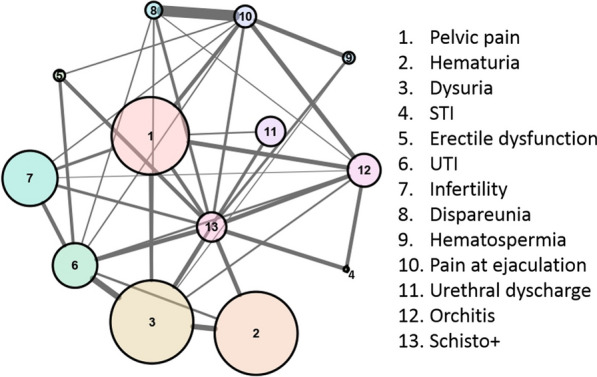

**Supplementary Information:**

The online version contains supplementary material available at 10.1186/s40249-024-01190-8.

## Background

Schistosomiasis is a major tropical disease caused by parasitic blood trematodes of the genus *Schistosoma* transmitted to humans through contact with fresh water infested with cercaria released from the intermediate snail host. *Schistosoma haematobium* infection is highly endemic throughout much of sub-Saharan Africa, affecting an estimated 112 million people [1, 2]. The infection is often acquired during childhood but reinfections are common and adult worms may remain present in the human body for up to 30 years [2–4]. If infection remains untreated, it becomes chronic several months after primary infection. Long-term chronic disease has a wide array of chronic non-specific clinical signs and symptoms and can lead to severe complications [5]. In non-endemic countries, schistosomiasis is mainly imported by travellers and recent migrants from endemic regions, with the chronic form being the most common presentation [6–8].

*Schistosoma haematobium* infects more specifically the urinary tract, where it can involve virtually any tissue and organ. Since the venous plexus of the urinary tract shares communications with the genitalia in both men and women, involvement of the genital systems can occur more frequently than commonly assumed [9]. Given the evidence of post-mortem and histopathological investigations and case reports [10–12], female and male genitourinary schistosomiasis (MGS) are often overlooked in patients, in spite of being described soon after recognition of the parasite [10].

In MGS, *Schistosoma* eggs passing through or becoming trapped in the tissues of the prostate, seminal vesicles, vas deferens, epididymis, or testes trigger immune reactions and granuloma formation [11], leading to morbidity and organ dysfunction, with all genital organs potentially affected [10–12]. Clinical features of MGS in endemic countries include genital or ejaculatory pain, haemospermia, infertility, and abnormally enlarged organs, as well as granulomatous infiltration, fibrosis, and calcifications [10–13].

Chronic *S. haematobium* infection is typically associated with a low parasite burden and its diagnostic workup is extremely challenging. Serum antibody tests cannot distinguish ongoing from past infection or measure parasite burden. In late chronic stages, serum antibody detection is much more sensitive than egg detection by microscopy, antigen detection, or even DNA sequences (PCR) in urine. Moreover, these urine techniques have been tested only in endemic countries and only in recently arrived migrants or travellers.

At the moment, schistosomiasis screening is recommended in non-endemic countries only for travellers and migrants who have been in endemic countries no more than 5 years previously [14]. The actual prevalence of infection in long-term migrants from endemic countries is largely unknown. Here we aim to characterize and assess the prevalence of unexplained male genitourinary signs and symptoms of MGS among long-term African migrant residents in Europe who have tested positive on a *Schistosoma* serology test.

## Subjects and methods

### Study design

Between April and November 2022, we carried out a multi-centre community-based cross-sectional study of African migrants living in two communities in Spain. The inclusion criteria were being a male, having been born in a schistosomiasis-endemic region of sub-Saharan Africa, living in Spain at the time of the study, and being over 18 years of age. The only exclusion criterion was awareness of having been previously diagnosed and treated for schistosomiasis.

### Study area and study population

The study sites comprised two municipalities (Mataró and Granollers) which have the largest pockets of sub-Saharan migrants in the North Metropolitan Barcelona health services district in Catalonia, Spain. This district serves around 1,300,000 inhabitants in the northern crown of the city of Barcelona, with up to 20,000 immigrants coming from sub-Saharan countries in 2021, mostly from Senegal (36.5%), Gambia (23.0%), and Mali (12%) [15], countries which had an estimated prevalence of schistosomiasis infection in adults in 2015 of 20.3%, 24.7% and 34.2%, respectively [16]. After a public awareness campaign carried out in the targeted migrant communities with the collaboration of cultural mediators and community leaders, we recruited consecutively all individuals who presented themselves voluntarily at local participating primary health care centres and signed a consent form.

### Data collection and management

We screened eligible participants for past and present clinical signs and symptoms related to the genitourinary and reproductive systems as classified in the International Classification of Diseases (11th Revision) [17]. We also collected selected laboratory findings (eosinophilia and liver transaminases) using two different sources: (1) the electronic clinical medical records database, which allows all primary health centres and hospitals in Catalonia to share data in a standardized format about all individuals registered with the public health care system; and (2) a structured questionnaire implemented by trained health personnel based on our previous experience in this area [18], and pretested prior to implementation of the study. We also used these two sources to gather socio-demographic data (age, years of residence, country of origin) and clinical genitourinary findings obtained without an etiological diagnosis (therefore excluding genitourinary symptoms attributed to other causes). Additional file 1: Table S1 lists and defines the 14 clinical signs and symptoms recorded. Two samples of whole blood per participant were subjected to basic haemogram and biochemical determination, serological testing for *Schistosoma* [Enzyme-Linked Immunosorbent Assay (ELISA-IgG) and immuno-chromatography (ICT IgG/IgM)]. A urine sample was also obtained at the time of each participant’s visit (between 10:00 AM and 2:00 PM) for analysis and microscopic examination to seek *S. haematobium* eggs. A test to detect the presence of eggs in stool was not performed. Data were introduced through the REDCap v. 11.0.3 (REDCap Consortium) secure web application.

### Laboratory procedures

A venous blood sample (5 ml) was collected and tested for *Schistosoma* spp. with the SCHISTO-96 test kit (Scimedx Corporation, Dover, USA). A spectrophotometer absorbance reading (450–620 nm) greater than 0.3 optical density (OD) units was regarded as a positive result for *Schistosoma* infection. Serum samples were assessed by two qualitative diagnostic serological tests, namely the ELISA for anti-schistosomiasis IgG antibodies (Euroimmun, Lübeck, Germany, ref. EI2300-9601G) and *Schistosoma* in-vitro ICT (LDBIO Diagnostics, Lyon, France, ref. BILZAbICT20), which simultaneously detects both IgM and IgG. Urine samples were processed by the sediMAX automatic sediment analyser with microscopy (77 Elektronika, Budapest, Hungary). For parasitological diagnosis, 10 ml were placed in a conical tube and centrifuged at 1000 *g* for 10 min to increase the sensitivity of the urine exam. Subsequently, 20 µl were placed on a slide for ova and parasite detection by optical microscopy.

### Statistical methods

Continuous variables were described in terms of mean and standard deviation (*SD*) or median and interquartile ranges (IQR) and categorical variables in terms of proportions and 95% confidence intervals (*CI*). Bivariate analysis used the Chi-square test or McNemar test for paired data, or Student’s t-test for categorical or continuous variables, respectively, or their nonparametric counterparts when necessary (Fisher test and Wilcoxon test) and multivariate analysis using logistic regression. Odds Ratios (*OR*) and corresponding 95% *CI* were estimated. We calculated a clinical score for each participant corresponding to the number of genital and reproductive tract signs and symptoms recorded. For the network and cluster analysis we regarded a case as indicating MGS when schistosomiasis tests were positive and clinical scores were over the estimated cut-off using the Youden (J) index. For this purpose, we performed a Receiving Operating Characteristics (ROC) analysis between schistosomiasis positivity and clinical score. Briefly, the estimated Youden index indicates the best sensitivity–specificity balance of a clinical score (cumulative number of clinical items) relative to schistosomiasis-positive test. We calculated the pairwise association between variables by means of Kendall’s *sb* test. Bonferroni’s method was used to account for multiple testing. Next, we built a network by connecting all variable pairs that had a significant association (corrected *p* value 0.1). Edges between any two variables were given a strength according to Kendall’s sb value for the pair. Data were analysed using Stata 14.0 (StataCorp LLC, College Station, Texas, USA) and R 3.1.4 (R Foundation for Statistical Computing, Vienna, Austria).

## Results

### General description

We enrolled a total of 388 eligible male participants, with a mean age of 43.5 years (*SD* = 12.0, range: 18–76). The median time since migration to the European Union was 17 (IQR: 11–21) years. However, most of them (72%) had visited their country of origin during the previous 5 years. The most frequent country of origin was Senegal (*N* = 179; 46.1%), followed by Gambia (*N* = 91; 23.5%). Overall, 147 (37.9%) were *Schistosoma*-positive as shown by an ELISA (*N* = 96; 24.7%) and/or ICT test (*N* = 112; 28.9%). *S. haematobium* eggs were observed in urine in 5/388 cases (1.3%). Seven participants (1.8%) had HIV infection, including 2 new diagnoses. Table 1 shows the descriptive data for the sample stratified by *Schistosoma* serology test positivity.

**Table 1:** Socio-demographic characteristics, laboratory results and HIV testing at enrolment of study participants, broken down by *Schistosoma* serology test result

		Total		Schisto + (*N* = 147)		Schisto– (*N* = 241)		
Variable	*N*^1^	*n*	%	*n*	%	*n*	%	*P*
*Age distribution (years)*								
18–25	388	31	8.0	10	*6.8*	21	8.8	*0.9*^*2*^
26–35		71	18.4	28	*19.1*	43	18.0	
36–45		130	33.7	51	*34.7*	79	33.1	
46–55		100	25.9	40	*27.2*	60	25.1	
> 55		54	14.0	18	*12.2*	36	15.1	
*Country of origin*	388							
Senegal		179	46.1	66	*44.9*	113	*46.9*	*0.7*
Gambia		91	23.5	25	*17.0*	66	*27.4*	*0.02*
Mali		61	15.7	30	*20.4*	31	*12.9*	*0.05*
Guinea–Conakry		30	7.7	14	*9.5*	16	*6.6*	*0.3*
Others		10	2.6	4	*2.7*	6	*2.5*	*0.6*
*Years living in EU*^*3*^	387							
< 5		45	11.6	16	*11.0*	29	*12.0*	*0.5*^*2*^
5 to 10		34	8.8	15	*10.3*	19	*7.9*	
10 to 15		53	13.7	16	*11.0*	37	*15.4*	
15 to 20		124	32.0	53	*36.3*	71	*29.5*	
> 20		131	33.9	46	*31.5*	85	*35.2*	
*Latest visit to country of origin*	372							
< 5 years		266	71.5	108	*77.1*	158	*68.1*	*0.06*
> 5 years or never		106	28.5	32	*22.9*	74	*31.9*	
*Swam in natural bodies of water while in country of origin*	265	48	18.1	20	*18.5*	28	*17.8*	*0.9*
*Laboratory results*								
Eosinophilia (> 5%)	378	106	28.0	46	31.9	60	*25.6*	*0.2*
Haematuria	377	84	22.3	35	24.3	49	*21.0*	*0.5*
Anaemia	379	85	22.4	34	23.6	51	*21.7*	*0.7*
Glomerular filtrate < 90	381	122	32.0	52	35.9	70	*29.7*	*0.2*
Creatinine > 1.2	383	54	14.1	19	13.1	35	*14.7*	*0.7*
Alanine aminotransferase > 50 U/L	382	12	3.1	3	2.1	9	*3.8*	*0.4*
Aspartate aminotransferase > 50 U/L	383	12	3.1	6	4.1	6	*2.5*	*0.4*
Alkaline phosphatase > 120 U/L	383	17	4.4	4	2.8	13	*5.5*	*0.2*
Gamma–glutamyl transferase > 55 U/L	382	54	14.1	26	17.9	28	*11.8*	*0.1*
Overall transaminase level	383	75	19.6	30	20.7	45	*18.9*	*0.7*
*Other coinfections*^*3*^								
HIV^4^	385	7	1.8	*5*	3.5	*2*	0.8	*0.07*

^1^Number of participants with data available; ^2^*P*-test for trend; ^3^European Union (EU); ^4^Human immunodeficiency virus

### Clinical features

**Fig. 1:**
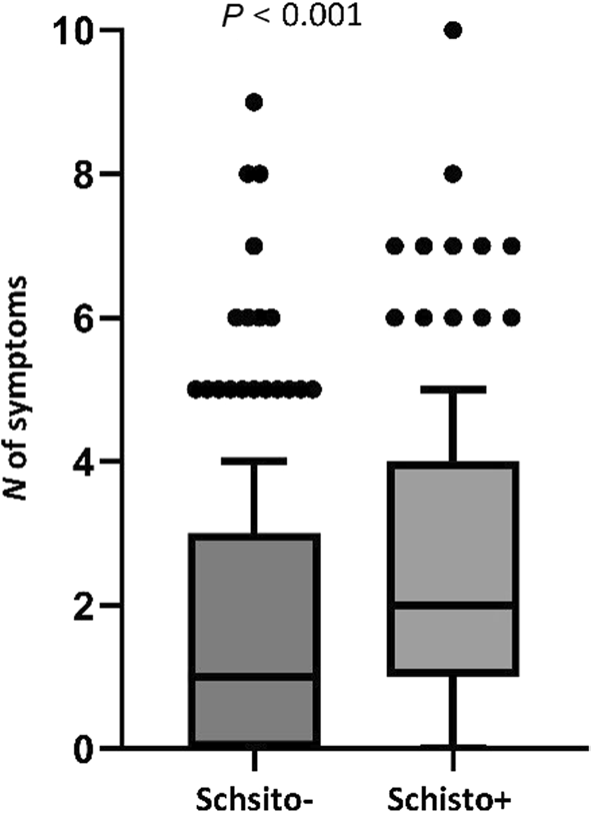
Box plot of number of genitourinary symptoms broken down by schistosomiasis serology test result. *N*: Number

Among the overall study sample, we found a median of two genital clinical signs or symptoms (IQR: 1–4, range: 0–10). Participants with a positive *Schistosoma* serology test result had a significantly higher number of genitourinary signs or symptoms: median of 2 (IQR: 1–4) vs. median of 1 (IQR: 0–3); *P* < 0.001 (Fig. 1).

We identified 9 out of the 14 clinical genitourinary variables (through either the questionnaire or clinical records) which were significantly associated with a positive schistosomiasis test result after adjusting for age. The most prevalent clinical findings within *Schistosoma*-positive individuals were history of haematuria (55.2%; *OR* vs. those with a negative serology = 2.37, 95% *CI*: 1.5–3.6), dysuria episodes (52.1%; *OR* = 2.01, 95% *CI*: 1.3–3.1), chronic recurrent pelvic pain (45.2%; *OR* = 1.57, 95% *CI*:1.0–2.4), self-reported infertility (32.1%; *OR* = 1.69, 95% *CI*: 1.0–2.8), and orchitis (20.7%; *OR* = 1.81, 95% *CI*; 1.0–3.1). The remaining signs or symptoms associated with a positive test were pain on ejaculation (14.5%; *OR* = 1.85, 95% *CI*: 1.0–3.5), erectile dysfunction (9.5%; *OR* = 3.10, 95% *CI*: 1.3–7.6), dyspareunia (12.4%; *OR* = 2.45, 95% *CI*: 1.2–5.2), and syndromic STI as defined in table S1 (5.4% vs. 0.8%; *P* = 0.008). However, eosinophilia was not more prevalent in subjects with a positive test result.

**Fig. 2:**
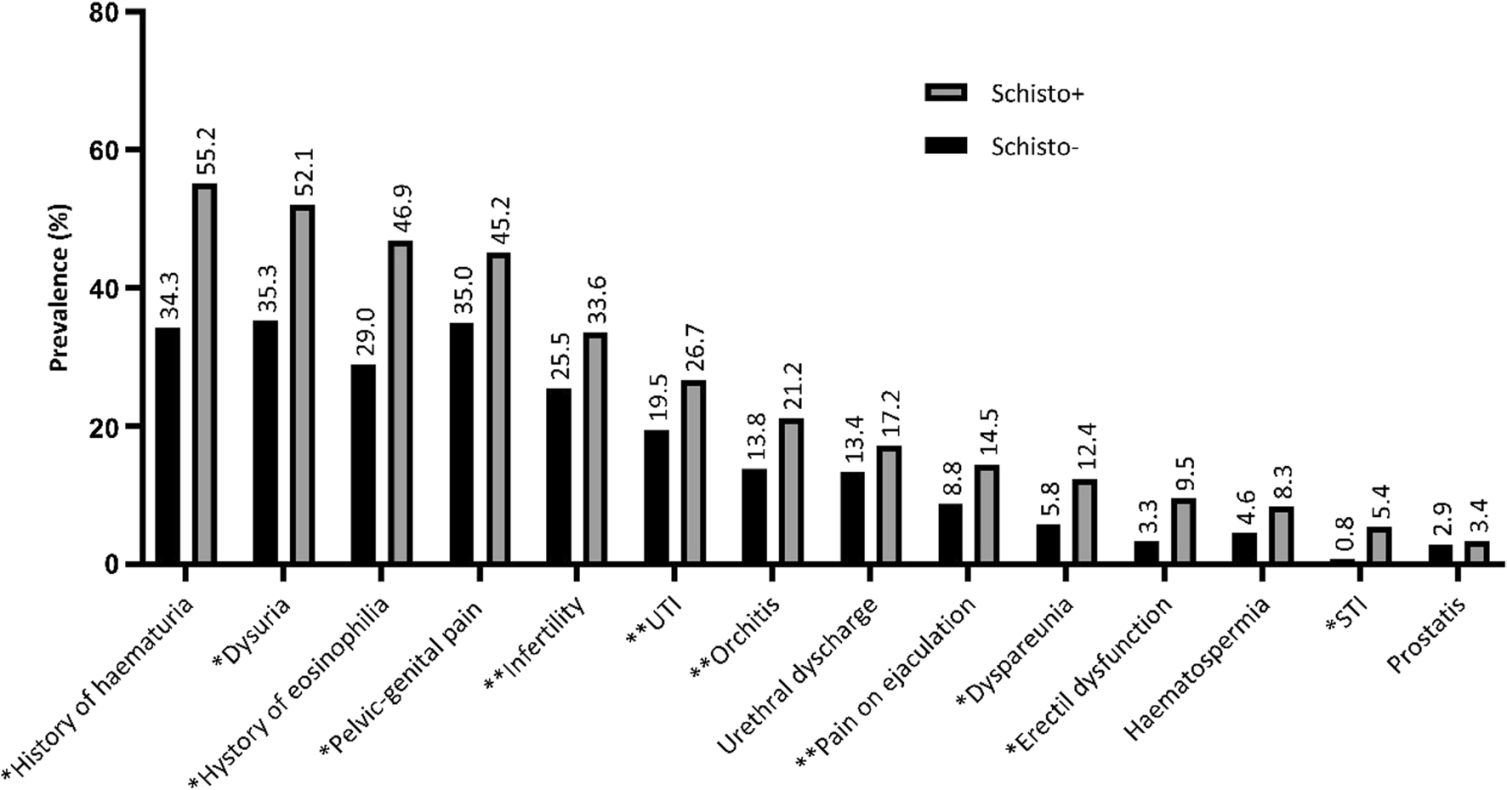
Prevalence of genitourinary signs and symptoms assessed in the study broken down by schistosomiasis serology test results^1^. ^1^We considered cumulative history of signs and symptoms obtained from both a directed questionnaire and clinical records. *P value < 0.05; **P value between 0.05 and 0.1. Add the abbreviations in this figure

Figure 2 shows the prevalence of clinical signs and symptoms assessed, broken down by schistosomiasis serology test result. Additional file 1: Table S2 shows the prevalence of signs and symptoms recorded from clinical history and questionnaire data, as well as totals with any overlap between history and questionnaire absorbed, and the corresponding *OR* adjusted by age. Overall, all items assessed with two sources of information were significantly captured through the directed questionnaire more frequently than from the search of electronic medical records, except syndromic Urine Tract Infections (UTI) (Additional file 1: Table S3).

### Clinical characterization and network analysis

**Fig. 3:**
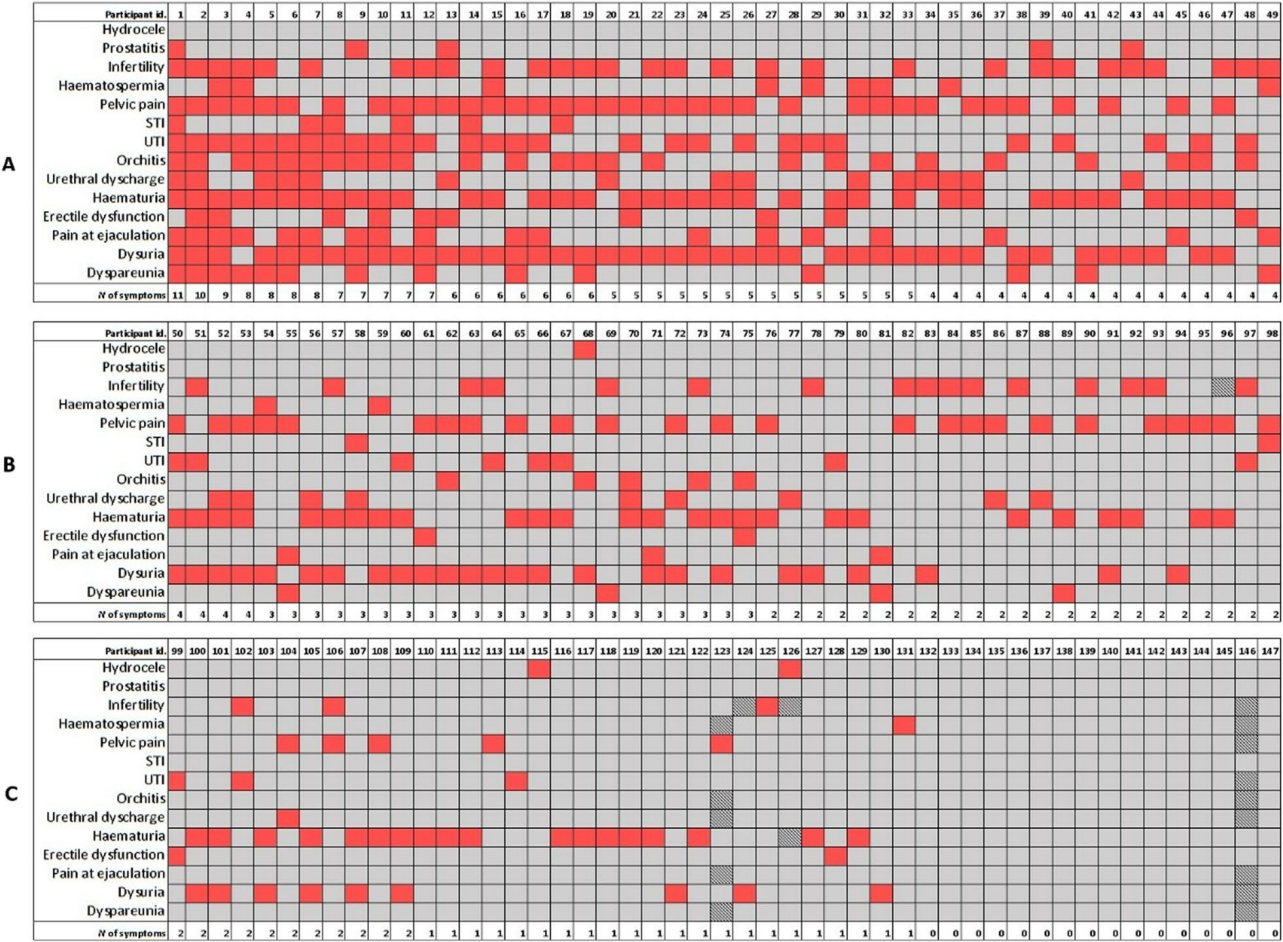
Accumulation of urogenital and reproductive system signs and symptoms among every participant in the study with a positive schistosomiasis serology test result (*N* = 147)^1^. ^1^Each column represents an individual with a positive schistosomiasis serology test result. The graphic representation has been ordered according to the cumulative number of clinical items in every case from box A (11 to 4), box B (4 to 2) to box C (2 to 0). The overall cumulative prevalence of each clinical item in decreasing order was: haematuria (*N* = 80, 55.2%), dysuria (*N* = 76; 52.1%), pelvic pain (*N* = 66, 45.2%), infertility (*N* = 48, 33.6%), orchitis (*N* = 30, 20.7%), pain on ejaculation (*N* = 21, 14.5%), dyspareunia (*N* = 18, 12.4%), erectile dysfunction (*N* = 14, 9.5%), haematospermia (*N* = 12, 8.3%), syndromic STI (*N* = 8, 5.4%), prostatitis (*N* = 5, 3.4%), and hydrocele (*N* = 3, 2.0%). Shaded squares indicates missing or unavailable data

Participants with schistosomiasis–positive serology tests (*N* = 147) showed multiple signs and symptoms (up to 11), as shown in Fig. 3. Among those with recent data available (< 6 months, *N* = 54), a majority had experienced recent episodes of abdominal pain (85.2%) or dysuria (61.1%) up to 6 months before enrolment, and 35.2% had experienced episodes of haematuria during adulthood. Three out of 58 (5.2%) had a semen exam performed during the workup of infertility, showing severe oligospermia, cryptozoospermia, or azoospermia. Anaemia and a glomerular filtration rate < 90 ml/min, increased transaminases, or any other lab measurement were not more common in people with a positive serology test result.

**Fig. 4:**
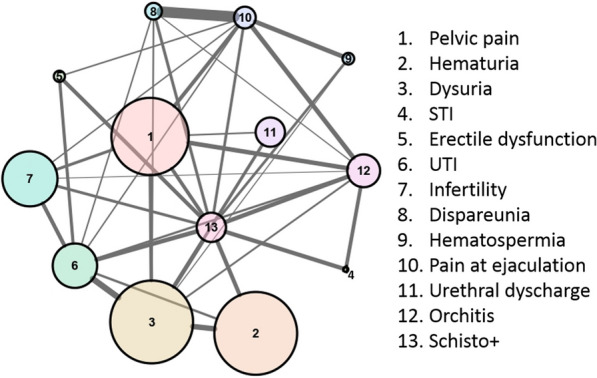
Network analysis of the signs and symptoms identified in the study sample*^1–3^. *Every clinical sign or symptom is a node. The size of the node correlates with the number of individuals presenting the given condition. An edge between nodes (variables) indicates a statistically significant association between them. The thickness of the edges indicates the strength of the association, which correlates with the number of times that both items appear together in the data set. A more central location of the node indicates a higher centrality score, which can be interpreted as a higher influence (higher density of connections) over the network. In Figure S1 we show the centrality scores of the network calculations for each of the variables examined. Briefly, the network suggests the existence of clusters of signs and symptoms around the case definition of genitourinary schistosomiasis used for this analysis (Schisto +). Note that the working case definition should not be considered a diagnostic criteria for MGS because it cannot be performed in the absence of reliable microbiological methods to identify parasites. ^1^UTI: Urinary tract infections; ^2^STI: Sexually Transmitted Infections. ^3^We considered for this analysis the cumulative history of signs and symptoms collected by means of both a directed questionnaire and a review of digitalized medical histories

The Youden index was estimated to be ≥ 3 symptoms, which was considered the best cut-off for sensitivity–specificity of a positive schistosomiasis serology test against genitourinary clinical manifestations. In total, 58 (14.9% of the whole sample) had a *Schistosoma*-positive serology test and a score over the estimated cut-off (39.5% of participants with a *Schistosoma*-positive test), and were regarded as the working case definition for the network analysis. This analysis, which included 10 nodes and 31 edges (Fig. 4), showed a specific pattern of clustering between the different signs and symptoms examined.

The analysis provided a higher centrality for the case definition including at least 3 clinical items, for all parameters measured, followed by dysuria and pain on ejaculation. Pain on ejaculation, dyspareunia, and haematospermia tended to present together in individuals, as did dysuria, haematuria, urethral discharge, and UTI, whereas infertility, pelvic pain, and erectile dysfunction tended not to cluster. To sum up, besides the high cumulative number of signs and symptoms among participants with positive serology test results, these symptoms tended to present not at random, but rather as clusters. (For a better interpretation of the network results, see footnote to Fig. 4.)

## Discussion

Our results show that, as a population, male long-term migrants from sub-Saharan African areas with a high endemicity of schistosomiasis tend to have a history of unexplained genitourinary signs and symptoms, which are significantly associated with having positive *Schistosoma* serology test results, even though urine exams fail to detect evidence of parasites. In most of the cases studied here, despite many previous contacts with the health care system in the receiving country during which no definitive diagnosis for those clinical signs or symptoms had been given, schistosomiasis had not been considered. In fact, only 11 over 399 (2.8%) individuals initially screened had received praziquantel in the past and were consequently excluded from this study. These data suggest that untreated long-lasting chronic infection with *S. haematobium* can be associated in males with a considerable burden of urogenital involvement and may be largely underestimated. A review of the literature found that all studies that focused exclusively on MGS were performed in endemic countries or only included recently arrived migrants [12]. In these studies symptoms associated with MGS were frequent (i.e. haematospermia and genital or coital pain), as was the observation of eggs in urine and/or semen [13, 19] or prostate biopsy [20]. However, studies were found that focused specifically on male MGS among long-term residents in non-endemic countries.

Whether these signs and symptoms correspond to low-grade persistent active infection or alternatively to post-infection chronic sequelae is currently unknown. The diagnosis of active schistosomiasis in people exposed so many years before is extremely difficult. The diagnostic yield of urine exams is low and urine antigen or even PCR tests have not been validated for chronic infections, nor are they routinely available in non–endemic countries. We found significantly higher rates of up to 8 key genitourinary clinical signs or symptoms in these untreated male sub-Saharan African migrants. These signs and symptoms included, in decreasing order of prevalence: haematuria, dysuria, pelvic pain, infertility, orchitis, dyspareunia, erectile dysfunction, and unspecific syndromic STI, all them without isolation of the causal agent. These conditions may have led to frequent consultations with the health care system and even misguided treatments for certain interrelated conditions.

According to our network analysis, male genitourinary signs and symptoms tend to present as clusters in any one individual rather than as a single clinical finding in isolation. Furthermore, the manifestations of chronic urogenital involvement of schistosomiasis may have different features compared to those seen in acute infection in recently exposed individuals. Our analysis indicates that the optimal definition of a potential MGS case is when at least 3 clinical signs or symptoms are present, for all parameters measured, followed by dysuria and pain on ejaculation. This suggests that these latter two are key symptoms of genitourinary schistosomiasis. We also found that pain on ejaculation, dyspareunia, and haematospermia tend to present together in individuals. By the same token, dysuria, haematuria, urethral discharge and UTI also tend to appear in clusters. In contrast, infertility, pelvic pain, and erectile dysfunction tend not to be clustered with other signs or symptoms.

The relationship to MGS of one characteristic condition, male infertility, has already been described in the context of endemic countries [12, 21]. There are different causal pathways underpinning infertility in schistosomiasis, including inflammation, mechanical blockage, the destruction of anatomical structures [21, 22], or hormonal disruption [23]. The prevalence of female infertility associated with *S. haematobium* infection in endemic countries seems to parallel the male infertility found in this study [24].

Overall, the weak ability of gold standard tests (i.e. direct urine or tissue microbiology determination of schistosomiasis) to confirm active disease among long-term migrants and the limited specificity and sensitivity of serologic methods [25, 26] accentuate the need for a set of clinical criteria to expedite screening for MGS in exposed migrants. It is important to also consider the linguistic and cultural factors that can impede access to health care in this migrant population [27, 28]. Directed questionnaires to identify occult genitourinary symptoms could be of help in this difficult scenario. In our study, the structured directed questionnaire identified a significantly higher prevalence of all occult genitourinary signs and symptoms (but not urinary tract infections) when actively sought than did clinical history records (Additional file 1: Table S2). This points to the sensitivity of structured questionnaires as a screening tool for chronic genitourinary schistosomiasis-associated signs and symptoms.

In contrast with the existing reported series, our study sample corresponded to individuals with long-lasting residence (median of 17 years) in a non-endemic European country, who had not been previously screened or were unaware of having been treated for schistosomiasis. While some endemic countries in sub-Saharan Africa have advanced schistosomiasis control or elimination programmes, others have yet to start programmes using the recommended strategies.

An earlier diagnosis would most likely have averted genitourinary involvement and chronic lesions in at least some of the cases studied here. Therefore, diagnostic delay is a matter of concern. The possible reasons for such delays include not only difficulties in accessing health care among migrant populations but also a lack of awareness among healthcare professionals about imported schistosomiasis and specifically the possibility of long-lasting urogenital disease associated with schistosomiasis. Importantly, the stigma surrounding male sexual and reproductive health among these communities deters many migrants from seeking care earlier for some of these symptoms. Additionally, the presence of non-genital manifestations of schistosomiasis, which are often more severe, may overshadow genital involvement in males, leading to underdiagnoses of MGS [12, 29]. The fact that the direct questionnaire employed in our study captured significantly more frequently nearly all items asked about indicates that this sort of instrument has the potential to prevent underdiagnoses or delayed diagnosis of this condition.

An additional consideration is the association reported between MGS and an increased susceptibility to HIV infection [30–32]. This is due to both overall immunological effects and local genital tract involvement with a friable epithelium and increased bleeding. Additionally, both diseases principally affect impoverished populations. In our study we identified a trend towards increased HIV incidence in men with a positive *Schistosoma* serology test (*P* = 0.07) and even diagnosed two new HIV cases. Screening for HIV should be considered for all potential long-term MGS patients.

Based on our results, it is clear that there are sufficient grounds to include schistosomiasis in the differential diagnosis of male African migrants with genitourinary signs and symptoms. A final question remains, however, Should empiric praziquantel treatment be automatically provided to individuals with suspected chronic genitourinary schistosomiasis? Considering the shortness of the two-dose treatment and its low toxicity and high efficacy in acute/recent infection, systematic praziquantel treatment might be advisable even when it is impossible to confirm the presence of active infection microbiologically. Given that an estimated 24% sub-Saharan Africans living in Europe (a population numbering around 4 million) [33] are thought to be schistosomiasis-positive [34], the benefits of such a strategy could be substantial, assuming that the treatment confirms its efficacy in controlled clinical trials. In this regard, a recent study highlighted that presumptive praziquantel treatment of all immigrants from endemic countries was an effective alternative in terms of costs, survival, and sequelae [35, 36]. However, the efficacy of praziquantel in chronic schistosomiasis with fibrotic lesions is uncertain [37–39]. MGS treatment studies with praziquantel have only been carried out in endemic countries, and in acute infections [22, 40, 41]. In addition, there is currently no high-grade evidence coming from randomized controlled trials comparing different treatment strategies for long-term schistosomiasis. The time elapsed since first infection, subsequent reinfections, age, and clinical presentation might all have a bearing on the most appropriate therapeutic approach as well. Given the potential severity of some of the genitourinary conditions—including severe and life-threatening complications like kidney insufficiency or bladder carcinoma—the administration of a standard two-dose praziquantel treatment should be explored as a potential strategy in suspected chronic MGS, once alternative diagnoses have been excluded and provided that the patient has tested positive for *Schistosoma* and comes from a high-endemic country.

Our study has certain limitations. As we have noted, there is a lack of a true gold standard to diagnose active infection in chronic MGS. Therefore, we cannot be categorically certain that all our participants had chronic active infection. Secondly, participants with more prominent symptoms or higher health-care awareness could well have been overrepresented in our series, while hard-to-reach populations might have been underrepresented due to the fact that inclusion in the study was voluntary. We also cannot exclude the possibility that some participants had received praziquantel many years ago in the context of mass drug distribution campaigns in their country of origin, something which they no longer recall or whose relevance to the present study participants did not recognize. However, given the long-term residence of most of them in Spain, the scope of such campaigns (if they took place) was likely too small to have affected them. An additional consideration is that serology-based testing cannot distinguish between *S. mansoni* and *S. haematobium* infection, whose respective geographical areas of distribution largely overlap in sub-Saharan Africa [16]. The ELISA test is reported to be less sensitive to *S. haematobium* [25], yet *S. haematobium* is by far the more frequent causative agent of MGS in African migrants. This may have diluted the associations we observed.

## Conclusions

This study identified a high prevalence of unexplained and interconnected genitourinary signs and symptoms, including positive *Schistosoma* serology test results but negative urine parasite exams, among long-term male migrants from areas of sub-Saharan African currently living in Spain. These signs and symptoms included significantly high rates of haematuria, dysuria, pelvic pain, infertility, orchitis, dyspareunia, erectile dysfunction, and syndromic STI, often associated with HIV infection. These results suggest that MGS may be more common than previously thought and greatly under-diagnosed in such populations. The lack of awareness of this condition among local primary care physicians, urologists, and STI clinics in non-endemic European countries, along with the stigma associated with genital conditions among male African migrants, may both help to explain the delay in diagnosis of this condition.

Screening programs for early diagnosis including structured directed questionnaires might be helpful to prevent long-term chronic MGS in this population. The effectiveness of treating imported MGS with praziquantel should also be studied. Further studies are also urgently needed to develop appropriate diagnostic tools to not only identify but also confirm the presence of persistent active infection in long-lasting chronic schistosomiasis, determine its true prevalence, and further characterize the clinical spectrum of male genitourinary involvement.

### Supplementary Information

**Additional file 1: Table S1.** Definition of genital signs and symptoms based on the ICD for which study participants were screened. **Table S2.** Prevalence of signs and symptoms among participants and their association with a positive *Schistosoma* serology test result. **Table S3.** Comparative analysis of prevalence of clinical signs and symptoms as recorded through the electronic clinical records search and as obtained through a direct screening questionnaire. **Figure S1.** Graphic representation of the centrality score of the variables included in the network analysis.

## Data Availability

Database may be made available upon formal request to and consent from the ethics board of reference (email: ceic.germanstrias@gencat.cat).

## Abbreviations

FGS: Female genital schistosomiasis
MGS: Male genital schistosomiasis
PCR: Polymerase chain reaction
ICD: International Classification of Diseases (11th Revision)
ELISA: Enzyme–linked immunosorbent assay
ICT: Immuno-chromatography
IQR: Interquartile range
*CI*: Confidence interval
*OR*: Odds ratio
ROC: Receiving operating characteristics
UTI: Urinary tract infections
STI: Sexually transmitted infections
HIV: Human immunodeficiency virus
