# Supplementary material for: Male genitourinary schistosomiasis-related symptoms among long-term Western African migrants in Spain: a prospective population-based screening study
Source: Infect Dis Poverty. 2024 Mar 7;13:23. doi: 10.1186/s40249-024-01190-8 (PMC10919049; doi:10.1186/s40249-024-01190-8)

**Table S1**: Definition of genital signs and symptoms screened in the study participants (International Classification of Diseases (10^TH^ Edition).

| **Clinical sign or symptom** | **Definition** | ICD-10 Code^1^ |
| --- | --- | --- |
| Erectil disfuntion or male erectyle disorder | *Self reported difficulty of getting and keeping an erection.* | *F52.2* |
| Haematospermia | *Macroscopic presence of blood in the semen.* | *N50.1* |
| Pain at ejaculation | *Self reported painful sensation occurring during orgasm.* | *N48.8* |
| Dyspareumia | *Recurrent or persistent genital or pelvic pain with sexual activity or sexual dysfunction that is present for three months or longer* | *N48.8* |
| Orchitis | *Presence of signs and symptoms of an inflammation (swelling, redness, pain, etc.) of one or both testicles* | *N45* |
| STI | *Unspecific syndromic sexually transmitted infection with negative microbiology workup, excluding characteristic or patognomonic presentations (i.e. Genital ulcer or chancroid)* | *A64* |
| Pelvic genital pain | *Recurrent or persistent genital or pelvic pain present for three months or longer* | *R10.3* |
| Dysuria | *Episode of pain or difficult urination* | *R30.9* |
| Urinary tract infection | *Syndromic presentation of signs and symptoms of urinary tract infection without isolation of pathogen* | *N39.0* |
| Infertility | *At least one episode of no-pregnancy after more than six months of sexual activity with the usual sexual partner without the use of contraception methods.* | *N46* |
| Uretrhal discharge | *Abnormal purulent or mucoid secretions from the penis* | *R36* |
| Spermal abnormalities | *Microscopic observation of spermal abnormalities (i.e. Oligospermia, azoospermia, etc.)* | *N46* |
| Prostatitis | *Clinical episode of prostate inflammation* | *N41.9* |
| Hidrocele/varicocele | *Swelling in the scrotum, the pouch of skin that holds the testicles* | *N43/I86.1* |
| Haematuria |  | *R31* |

- - - 1. Available at https://icd.who.int/browse10/2019

**Table S2:** Prevalence and association of examined signs and symptoms among participants with a positive Schistosoma serology

| Clinical sign/symptom | *N*/total | *%* | *N*/Sch+ | *%* | *OR*^1^ | 95%*CI* | *p* |
| --- | --- | --- | --- | --- | --- | --- | --- |
| Erectile dysfunction | *22/388 | 5.7 | 14/147 | 9.5 | 3.10 | (1.3-7.6) | 0.01 |
| Haematospermia | *3/377 | 0.8 | 2/144 | 1.4 | --- | --- | 0.3^2^ |
|  | ǂ23/385 | 6.0 | 12/145 | 8.3 | 1.87 | (0.8-4.4) | 0.1 |
|  | §23/385 | 6.0 | 12/145 | 8.3 | 1.87 | (0.8-4.4) | 0.1 |
| Pain on ejaculation | *4/377 | 1.1 | 4/144 | 2.8 | --- | --- | 0.02^2^ |
|  | ǂ41/385 | 10.6 | 20/145 | 13.8 | 1.75 | (0.9-3.4) | 0.1 |
|  | §42/385 | 10.9 | 21/145 | 14.5 | 1.85 | (1.0-3.5) | 0.07 |
| Dyspareunia | *3/376 | 0.8 | 2/144 | 1.4 | --- | --- | 0.3^1^ |
|  | ǂ31/385 | 8.1 | 18/145 | 12.4 | 2.67 | (1.2-5.7) | 0.01 |
|  | §32/385 | 8.3 | 18/145 | 12.4 | 2.45 | (1.2-5.2) | 0.02 |
| Orchitis | *16/377 | 4.2 | 5/144 | 3.5 | 0.96 | (0.3-2.7) | 0.9 |
|  | ǂ60/385 | 15.6 | 28/145 | 19.3 | 1.67 | (0.9-2.9) | 0.08 |
|  | §63/385 | 16.4 | 30/145 | 20.7 | 1.81 | (1.0-3.1) | 0.04 |
| Sexually Transmitted Infections (STIs) | *10/388 | 2.6 | 8/147 | 5.4 | --- | --- | 0.008^2^ |
| Pelvic genital pain | *23/377 | 6.1 | 15/144 | 10.4 | 3.74 | (1.5-9.4) | 0.005 |
|  | ǂ148/385 | 38.4 | 64/145 | 44.1 | 1.50 | (1.0-2.3) | 0.06 |
|  | §150/386 | 38.9 | 66/146 | 45.2 | 1.57 | (1.0-2.4) | 0.04 |
| Dysuria | *55/379 | 14.5 | 28/144 | 19.4 | 1.84 | (1.0-3.3) | 0.04 |
|  | ǂ146/387 | 37.7 | 70/146 | 47.9 | 2.02 | (1.3-3.1) | 0.001 |
|  | §161/387 | 41.6 | 76/146 | 52.1 | 2.01 | (1.3-3.1) | 0.001 |
| Urinary tract infection | *51/379 | 13.5 | 22/144 | 15.3 | 1.28 | (0.7-2.3) | 0.4 |
|  | ǂ54/387 | 14.0 | 24/146 | 16.4 | 1.43 | (0.8-2.6) | 0.2 |
|  | §86/387 | 22.2 | 39/146 | 26.7 | 1.53 | (0.9-2.5) | 0.09 |
| Infertility^2^ | *59/378 | 15.6 | 27/143 | 18.9 | 1.47 | (0.8-2.6) | 0.2 |
|  | ǂ84/326 | 25.8 | 42/131 | 32.1 | 1.69 | (1.0-2.8) | 0.04 |
|  | §108/378 | 28.6 | 48/143 | 33.6 | 1.47 | (0.9-2.3) | 0.1 |
| Urethral discharge | *5/377 | 1.3 | 3/144 | 2.1 | --- | --- | 0.3 ^2^ |
|  | ǂ55/384 | 14.3 | 24/145 | 16.6 | 1.32 | (0.7-2.3) | 0.4 |
|  | §57/384 | 14.8 | 25/145 | 17.2 | 1.38 | (0.8-2.5) | 0.3 |
| Sperm abnormalities | *9/388 | 2.3 | 4/147 | 2.7 | --- | --- | 0.5^2^ |
| Prostatitis | *12/388 | 3.1 | 5/147 | 3.4 | 1.17 | (0.4-3.8) | 0.8 |
| Hydrocele/varicocele | *6/388 | 1.5 | 3/147 | 2.0 | --- | --- | 0.4^2^ |
| *Other non-genital findings* |  |  |  |  |  |  |  |
| History of haematuria | 161/381 | 42.3 | 80/145 | 55.2 | 2.37 | (1.5-3.6) | <0.001 |
| History of eosinophilia | 137/383 | 35.8 | 68/145 | 46.9 | 2.42 | (0.8-7.8) | 0.1 |

*From clinical records; ǂ From questionnaire ; § Total with overlap absorbed. 1. Odds ratio of prevalence in people with a positive serology vs those with a negative serology, adjusted by age 2. Fisher’s exact test.

**Table S3**: Comparative analysis of prevalence of clinical signs and symptoms recorded through the electronic clinical records search and through a direct questionnaire screening.

|  | **Clinical records** | | | **Questionnaire-based** | |  |
| --- | --- | --- | --- | --- | --- | --- |
| **Clinical variable** | *N*^1^ | *n*^2^ | *%* | *n*^2^ | *%* | *p*^3^ |
| Erectile disfuntion^4^ | 388 | 22 | *5.7* | --- | *---* | --- |
| Haematospermia | 376 | 3 | *0.8* | 23 | *6.1* | <0.001 |
| Pain at ejaculation | 376 | 4 | *1.1* | 40 | *10.6* | <0.001 |
| Dyspareumia | 375 | 3 | *0.8* | 31 | *8.3* | <0.001 |
| Orchitis | 376 | 16 | *4.3* | 58 | *15.4* | <0.001 |
| Pelvic genital pain | 376 | 22 | *5.9* | 144 | *38.3* | <0.001 |
| Dysuria | 380 | 56 | *14.7* | 145 | *38.2* | <0.001 |
| UTI | 380 | 51 | *13.4* | 54 | *14.2* | 0.8 |
| Infertility | 321 | 40 | *12.5* | 83 | *25.9* | <0.001 |
| Urethral discharge | 375 | 5 | *1.3* | 56 | *14.9* | <0.001 |
| Prostatitis^5^ | 388 | 12 | *3.1* | --- | *---* | --- |
| Hidrocele/varicocele^5^ | 388 | 6 | *1.5* | --- | *---* | --- |
| Haematuria | 367 | 37 | *10.1* | 112 | *30.5* | <0.001 |

1. Number of participants with data available from both sources. 2. Number of participants with the variable registered (clinical record) or self-referred (questionnaire). 3 McNemar test for paired data. 4. Only questionnaire-based data was available. 5. Medical terms, only clinical-records based data available.

**Figure S1**: Graphical representation of the centrality score (strength) of the variables included in the network analysis (Figure 4)


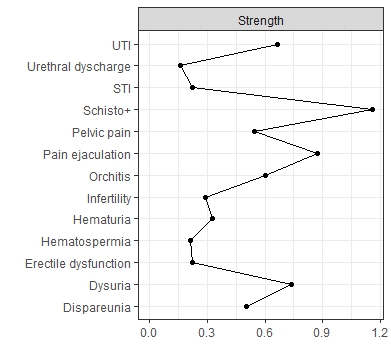

Supplement: Supplementary file 1 — Additional file 1: Table S1. Definition of genital signs and symptoms based on the ICD for which study participants were screened. Table S2. Prevalence of signs and symptoms among participants and their association with a positive Schistosoma serology test result. Table S3. Comparative analysis of prevalence of clinical signs and symptoms as recorded through the electronic clinical records search and as obtained through a direct screening questionnaire. Figure S1. Graphic representation of the centrality score of the variables included in the network analysis. [file 40249_2024_1190_MOESM1_ESM.docx]
